# Supplementary material for: Association of Urinary and Plasma Levels of Trimethylamine N-Oxide (TMAO) with Foods
Source: Nutrients. 2021 Apr 23;13(5):1426. doi: 10.3390/nu13051426 (PMC8145508; doi:10.3390/nu13051426)
Supplement: Supplementary file 1 [file nutrients-13-01426-s001.zip › nutrients-1180114-supplementary.pdf]

Table S1. Methodology for TMAO determination

Methodology for TMAO determination	Description	Ref.
LC-ESI-MS/MS	Liquid chromatographic electrospray ionization. A fast liquid chromatography tandem mass spectrometry (LC-MS/MS) method that can analyze TMAO in three minutes.	1
LC /MS	Stable isotope dilution liquid chromatography – tandem MS	2
LC-MS/MS	Liquid chromatography coupled with tandem MS	3
MSI-CE-MS	Multisegment injection-capillary electrophoresis-MS	4
NMR	Automated nuclear magnetic resonance spectroscopy assay	5
TMA/DMA	Valuation of the increase in trimethylamine after reduction of the sample (2 ml) with aqueous titanous chloride	6
UHPLC-MS/MS	Ultra-high performance liquid chromatography-MS combined with tandem MS	7
UPLC-ESI-MS/MS	Ion-pairing chemistry based ultra-performance liquid chromatography-electrospray ionization-tandem MS	8

MS: mass spectrometry (MS)

Reference:

1. van der Laan T, Kloots T, Beekman M, Kindt A, Dubbelman AC, Harms A, van Duijn CM, Slagboom PE, Hankemeier T. Fast LC-ESI-MS/MS analysis and influence of sampling conditions for gut metabolites in plasma and serum. Sci Rep. 2019 Aug 26;9(1):12370. doi: 10.1038/s41598-019-48876-w. PMID: 31451722; PMCID: PMC6710273.
2. Wang Z, Levison BS, Hazen JE, Donahue L, Li XM, Hazen SL (2014) Measurement of trimethylamine-N-oxide by stable isotope dilution liquid chromatography tandem mass spectrometry. Anal Biochem 455:35–40. https://doi.org/10.1016/j.ab.2014.03.016
3.Gessner A, di Giuseppe R, Koch M, Fromm MF, Lieb W, Maas R. Trimethylamine-N-oxide (TMAO) determined by LC-MS/MS: distribution and correlates in the population-based PopGen cohort. Clin Chem Lab Med. 2020 Apr 28;58(5):733-740. doi: 10.1515/cclm-2019-1146. PMID: 32084001.
4. Drouin N, Kloots T, Schappler J, Rudaz S, Kohler I, Harms A, Lindenburg PW, Hankemeier T. Electromembrane Extraction of Highly Polar Compounds: Analysis of Cardiovascular Biomarkers in Plasma. Metabolites. 2019 Dec 18;10(1):4. doi: 10.3390/metabo10010004. PMID: 31861366; PMCID: PMC7022788.
5. Garcia E, Wolak-Dinsmore J, Wang Z, Li XS, Bennett DW, Connelly MA, Otvos JD, Hazen SL, Jeyarajah EJ. NMR quantification of trimethylamine-N-oxide in human serum and plasma in the clinical laboratory setting. Clin Biochem. 2017 Nov;50(16-17):947-955. doi: 10.1016/j.clinbiochem.2017.06.003. Epub 2017 Jun 15. PMID: 28624482; PMCID: PMC5632584.
6. Zhang AQ, Mitchell SC, Smith RL. Dietary precursors of trimethylamine in man: a pilot study. Food Chem Toxicol. 1999 May;37(5):515-20. doi: 10.1016/s0278-6915(99)00028-9. PMID: 10456680.
7. Awwad, H. M., Geisel, J. & Obeid, R. Determination of trimethylamine, trimethylamine N-oxide, and taurine in human plasma and urine by UHPLC–MS/MS technique. J. Chromatogr. B 1038, 12–18 (2016).
8. Bhandari D, Bowman BA, Patel AB, Chambers DM, De Jesús VR, Blount BC. UPLC-ESI-MS/MS method for the quantitative measurement of aliphatic diamines, trimethylamine N-oxide, and β-methylamino-l-alanine in human urine. J Chromatogr B Analyt Technol Biomed Life Sci. 2018 Apr 15;1083:86-92. doi: 10.1016/j.jchromb.2018.02.043. Epub 2018 Mar 2. PMID: 29524697; PMCID: PMC7919444.
